# Preliminary performance of the VIDAS TB-IGRA as an aid in the diagnosis of individuals infected with *Mycobacterium tuberculosis*

**DOI:** 10.1128/jcm.01641-24

**Published:** 2025-05-14

**Authors:** Potiandi Serge Diagbouga, Arthur Diakourga Djibougou, Camille Pease, Ariana Alcaide, Audrey Berthoux, Natalie Bruiners, Daniela Maria Cirillo, Ardjouma Combary, Nadine Falchero, Deborah Handler, Antoinette Kaboré, Alfred Lardizabal, Amanda Lopes, Marissa Loubet, Philippe Manivet, Clemence Margain, Valerie Meunier, Faiza Mougari, Alberta Onyuka, Sophie Rivoiron, Tani Sagna, Mathilde Sanvert, Léon Sawadogo, Jacques Simporé, Emmanuelle Cambau, Maria Laura Gennaro

**Affiliations:** 1 Institut de Recherche en Sciences de la Santé, Centre National de la Recherche Scientifique et Technologique (IRSS/CNRST) 206843, Ouagadougou, Burkina Faso; 2 Department of Research and Development for Immunoassays, bioMerieux 1896 , Marcy-l'Étoile, Auvergne-Rhône-Alpes, France; 3 Global Tuberculosis Institute, Rutgers New Jersey Medical School 214907, Newark, New Jersey, USA; 4 Public Health Research Institute, Rutgers New Jersey Medical School 214907, Newark, New Jersey, USA; 5 Department of Medicine, Rutgers New Jersey Medical School, Newark, New Jersey, USA; 6 IRCCS San Raffaele Scientific Institute, Milan, Italy; 7 Programme national de lutte contre la tuberculose, Ouagadougou, Burkina Faso; 8 Internal Medicine Department, Département Médico-Universitaire INVICTUS, Lariboisière Hospital, Assistance Publique-Hôpitaux de Paris (APHP), Nord-Université Paris-Cité, Paris, France; 9 APHP, Biobank Lariboisière BB-0033-00064, Platform of BioPathology and Innovative Technologies in Health, Hôpital Lariboisière 378772, Paris, Île-de-France, France; 10 Mycobacteriology Laboratory APHP-Nord associated to French National Reference Center for Mycobacteria, IAME, University Paris Cite, Paris, France; 11 Centre de recherche biomoléculaire Pietro Anigoni (CERBA), Ouagadougou, Burkina Faso; The University of North Carolina at Chapel Hill School of Medicine, Chapel Hill, North Carolina, USA

**Keywords:** *Mycobacterium tuberculosis* complex, IGRA, VIDAS, QFT-Plus

## Abstract

This preliminary study compares VIDAS TB-IGRA (bioMérieux, Marcy-l'Etoile, France) with the established QuantiFERON-TB Gold Plus (QFT-Plus) (Qiagen, Hilden, Germany) to evaluate diagnostic performance for the diagnosis of individuals infected with *Mycobacterium tuberculosis* complex (latent infection and disease). The study was multi-center and performed between 2 October 2019 and 4 February 2020. Participants were divided into tuberculosis (TB) disease, high-risk, and low-risk populations. The confirmed TB disease population included 104 patients. The high-risk population included 162 individuals with flagged risk factors on a questionnaire but without objective clinical confirmation of TB. The low-risk population included 117 healthy blood donors from the French National Blood Bank. Positive and negative percent agreement (PPA and NPA) were determined between the VIDAS TB-IGRA and QFT-Plus. In the TB disease population, sensitivity was measured against bacterial culture and PCR. The VIDAS TB-IGRA produced fewer indeterminate results than the QFT-Plus (1/104 vs 23/104) in the TB disease population and exhibited a sensitivity of 95.0% against bacterial culture. Furthermore, a 98.2% PPA was obtained in comparison to QFT-Plus. In the low-risk population, the VIDAS TB-IGRA demonstrated high specificity (94.9%) and a strong NPA (98.2%) compared to QFT-Plus. In the high-risk population, the VIDAS TB-IGRA exhibited a strong PPA (94.4%) with the QFT-Plus. A lower NPA was observed (85.2%) compared to QFT-Plus, which may be due to a higher sensitivity demonstrated in the TB disease population. The fully automated VIDAS TB-IGRA is a promising aid in the diagnosis of individuals infected with *Mycobacterium tuberculosis* (latent infection and active disease). It exhibits higher sensitivity while maintaining specificity and produces fewer indeterminate interpretations than QFT-Plus. Its easy-to-use, single-patient format may lead to increased TB testing to aid in the adequate diagnosis and management of the disease.

**IMPORTANCE:**

This study presents a comprehensive evaluation of the VIDAS TB-IGRA diagnostic test. This test is compared with the established QuantiFERON-TB Gold Plus to assess its effectiveness in diagnosing both latent and active tuberculosis (TB) infections. The study highlights the VIDAS TB-IGRA’s higher sensitivity, fewer indeterminate results, and robust performance across different patient populations, including those with confirmed TB disease, high-risk, and low-risk groups. The findings suggest that the VIDAS TB-IGRA could enhance TB diagnosis and management, offering a fully automated, easy-to-use solution that reduces human error and result variability.

## INTRODUCTION

Tuberculosis (TB) is a disease caused by a bacterial pathogen (*Mycobacterium tuberculosis*) that most often affects the lungs. Globally, it is one of the leading causes of death from a single infectious agent (1). According to the World Health Organization, 1.25 million individuals died from TB, and approximately 10.8 million became ill with the disease in 2023 (2). Furthermore, approximately one-fourth of the global population is estimated to have been infected with TB bacilli. Among them, there is a 5%–10% risk of becoming ill and potentially spreading the disease within their lifetime. Individuals with latent TB infection do not exhibit symptoms and cannot transmit the disease. TB is preventable and treatable. However, the number of cases being properly diagnosed and treated remains low (1). Therefore, the quick and accurate detection of both *M. tuberculosis* infection and disease is essential to adequately control this pandemic.

Currently used diagnostic methods for latent TB infection and disease include bacterial culture, polymerase chain reaction (PCR), chest radiography, the tuberculin skin test (TST), and interferon-γ release assays (IGRAs). Bacterial culture is the current gold standard for the diagnosis of TB disease, followed by PCR, whereas the TST and IGRAs are the most commonly used methods to diagnose a latent TB infection. The TST has been available and widely used since the 1900s. It uses a heat-killed purified protein derivative from *M. tuberculosis* cultures, which shares components with other mycobacteria. Therefore, the TST cannot distinguish between *M. tuberculosis* infection and reactivity due to previous vaccination with the Bacillus Calmette-Guerin (BCG) vaccine or infections caused by non-tuberculous mycobacteria (NTM) (1, 3, 4). This may change with the development of new *Mycobacterium tuberculosis* antigen-based skin tests (5).

Since 2001, the development of IGRAs has provided many benefits over the TST for the detection of TB latent infection. The QuantiFERON-TB Gold Plus (QFT-Plus, Qiagen, Hilden, Germany) and T-SPOT-TB (Oxford Immunotec, Abingdon, UK) have been the most widely used, FDA-approved IGRAs that utilize synthetic peptides derived from *M. tuberculosis-*specific early secreted antigenic target 6 (ESAT-6) and culture filtrate protein 10 (CFP-10). These tests are used to detect *ex vivo* reactivity specifically associated with *M. tuberculosis* infection. False-positive results due to BCG cross-reaction are very low with these IGRAs because the synthetic peptides used are absent in BCG vaccine strains and most pathogenic NTM strains. Additionally, these IGRAs only require a single patient visit, and results can be made available within 24 hours of testing, unlike the TST, which requires a second visit within 48–72 hours.

Although the development of IGRAs represents a huge breakthrough in diagnosing latent TB infection, many drawbacks remain. The currently available IGRAs involve multiple cumbersome manual steps, which can increase the likelihood of human error and introduce variability in the execution of the preanalytical steps, potentially leading to erroneous results and interpretations (6–8). Additionally, these IGRAs are typically performed as batch tests that require several patient samples per microplate, which may delay results for days.

The newly developed, fully automated VIDAS TB-IGRA offers an improved method for the detection of both latent TB infection and disease. The fully automated system uses the commercially available VIDAS3 platform to quickly deliver results in one easy step, thus eliminating the multiple steps required by other IGRAs, decreasing the occurrence of human (or operator) error, and enhancing robustness with lower variability in results. Furthermore, the process is performed in a single-patient format, which allows for a quick (within 17 hours) turnaround time.

In the present study, we evaluated and compared the first preliminary performance of VIDAS TB-IGRA with that of the QFT-Plus to detect both latent TB infection and disease in three patient populations, including individuals with confirmed TB disease as well as high-risk or low-risk populations for latent TB infection. Our data demonstrate an improved detection capacity with maintained specificity for the optimized VIDAS TB-IGRA, suggesting its value in diagnosing both latent TB infection and disease.

## MATERIALS AND METHODS

### Participants

**Fig 1:**
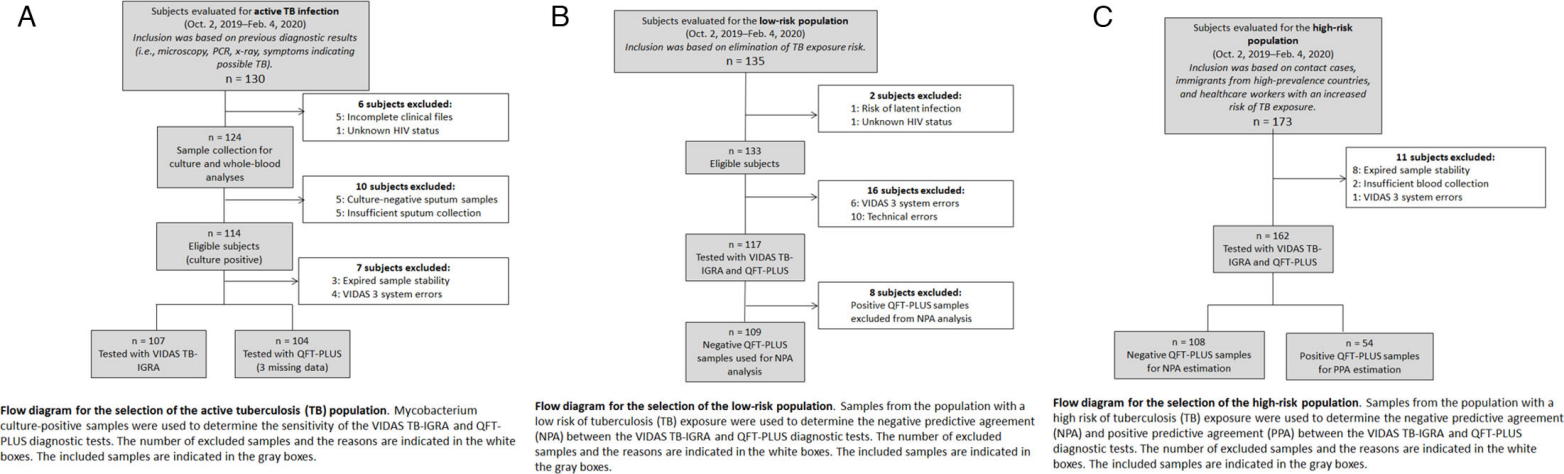
Patient study inclusion criteria. (A) Flow diagram for the inclusion of TB disease population. (B) Flow diagram for the inclusion of low-risk population. (C) Flow diagram for the inclusion of high-risk population.

Participants were divided into TB disease, high-risk, and low-risk populations according to the eligibility criteria outlined in Fig. 1A through C. TB disease was determined by bacterial culture or PCR. All study participants in the TB disease group were ≥2 years of age and were HIV negative.

The high-risk population consisted of patients who had an increased risk of *M. tuberculosis* exposure or infection based on a patient questionnaire. Individuals were considered to be at high risk of TB infection if they lived with someone with TB disease, lived in or emigrated from a country with a high prevalence of TB disease, spent more than 1 month in an area with a high prevalence of TB infection, belonged to a group with high TB transmission rates (e.g., homeless, incarcerated, or previously incarcerated individuals and intravenous drug users), abused drugs and/or alcohol, worked or resided at facilities with high-risk individuals (e.g., hospitals, homeless shelters, correctional facilities, nursing homes, and HIV patient residences), were children or adolescents exposed to high-risk adults, or were chronically immunocompromised or on immunosuppressive treatments. All study participants in the high-risk population were ≥2 years of age.

The low-risk population consisted of individuals who were previously questioned concerning their history of TB and accepted by the French National Blood Bank as healthy blood donors. All study participants of the low-risk population were ≥18 years of age.

Individuals who had received >15 days treatment for ongoing TB disease, received anti-TNF-α treatment within the past 3 months, had a positive TST within the past 12 weeks, had been diagnosed with an NTM infection, or had been diagnosed with HIV at the time of inclusion or 1 month prior were excluded from the study. Pregnant women were also excluded from the study. Additional patient information was collected for data monitoring and follow-up (Table S1). Written informed consent was obtained from all study participants.

### Sample collection and diagnostic testing

Two whole-blood samples were collected from each participant into 4 mL lithium heparin blood collection tubes. One sample was used for the VIDAS TB-IGRA test, and the other was used for the QFT-Plus test.

Samples were obtained and tested from 2 October 2019 to 4 February 2020 at the following four sites: Institut de Recherche en Sciences de la Santé (IRSS), Ouagadougou, Burkina Faso; Rutgers New Jersey Medical School, Newark, NJ, USA; Hôpital Lariboisière AP-HP/Assistance Publique-Hopitaux de Paris, France; and samples obtained from the French National Blood Bank (EFS) were tested at bioMérieux Marcy L’Etoile, Rhone-Alpes, France. Samples were routinely stored either at 2°C–8°C for ≤32 hours or at 18°C–25°C for ≤6 hours prior to testing.

The VIDAS TB-IGRA and QFT-Plus (blood collection tubes and microplate ELISA) diagnostic tests were simultaneously performed for each sample, according to the approved VIDAS TB-IGRA protocol (see below) and the QFT-Plus manufacturer’s instructions. For the VIDAS TB-IGRA, each sample was automatically subdivided into a negative control (NIL) sample, an antigen response sample (AG-NIL), and a mitogenic response positive control (MIT-NIL) sample. For the QFT-Plus, each sample was subdivided into a negative control (Nil) sample, two antigen response samples (TB1 and TB2), and a mitogenic response positive control (Mitogen) sample. Sample interferon-γ (IFN-γ) values were used to obtain a positive, negative, or indeterminate interpretation based on the established thresholds for each method.

### VIDAS TB-IGRA

**Fig 2:**
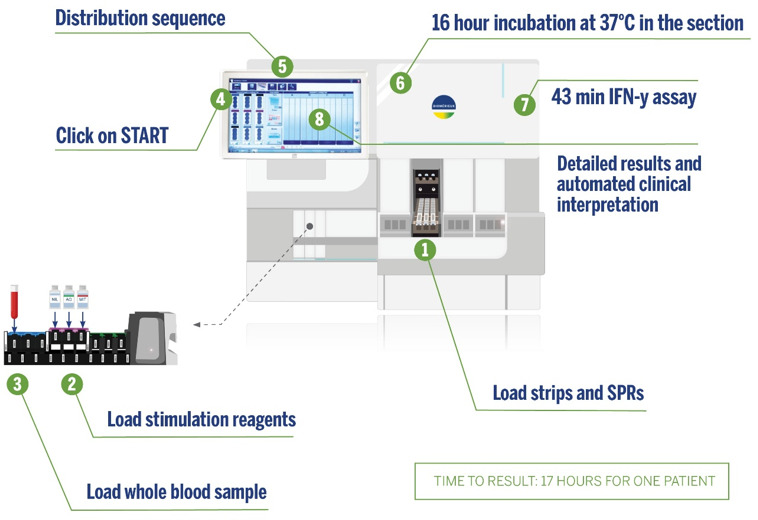
VIDAS TB-IGRA workflow.

The VIDAS TB-IGRA test was performed using the supplied reagents and the VIDAS3 system (Fig. 2). Each whole-blood sample was tested using three VIDAS reagent strips and three solid phase receptacles. The samples were loaded into the instrument with the three stimulation reagents (AG, antigen; MIT, positive control; and NIL, negative control). The antigens present in the AG stimulation reagent are derived from the *M. tuberculosis* proteins ESAT-6 (6 kDa) and CFP-10. These proteins are shared by all species of the *M. tuberculosis* complex (*M. tuberculosis*, *Mycobacterium bovis*, *Mycobacterium africanum*, *Mycobacterium microti*, *Mycobacterium canetti*, and *Mycobacterium caprae*) while absent in all BCG strains and in most NTM species. The MIT reagent is constituted by a plant-derived lectin in a formulated buffer. Reagent transfers were performed by the VIDAS3 automatic pipetting unit. The samples were homogenized and incubated with the individual stimulation reagents for 16 hours at 37°C in the strips. Next, the automated system performed an enzyme-linked immunofluorescent assay to quantify the sample IFN-γ concentrations. The final results were interpreted using internal calculation tools and previously established thresholds (Table 1).

**TABLE 1:** VIDAS TB-IGRA threshold values

NIL (IU/mL)	AG-NIL (IU/mL)	MIT-NIL (IU/mL)	Interpretation	Clinical significance
<6.4	>0.35 and >25% NIL	Any	Positive	*M. tuberculosis* infection likely
	<0.35 or >0.35 and <25% NIL	>1.1	Negative	*M. tuberculosis* infection not likely
	<0.35 or >0.35 and <25% NIL	<1.1	Indeterminate MIT low	Likelihood of *M. tuberculosis* infection cannot be determined
>6.4	Any		Indeterminate NIL high	

Briefly, AG-NIL IFN-γ concentrations ≥0.35 IU/mL indicated a positive result, whereas concentrations ≤0.35 IU/mL indicated a negative result. Results were considered indeterminate if MIT-NIL IFN-γ concentrations were <1.1 IU/mL or if NIL IFN-γ concentrations were ≥6.4 IU/mL.

### Statistical analyses

To establish statistical significance between the results of the QFT-Plus and VIDAS TB-IGRA tests in the TB disease population, data were arranged into a contingency table, and McNemar’s test was applied. A *P*-value < 0.05 was considered statistically significant. Negative percent agreement (NPA) and positive percent agreement (PPA) were calculated to evaluate the performance of the VIDAS TB-IGRA compared with that of the QFT-Plus. A Wilcoxon signed-rank test was conducted to determine whether there was a significant difference in median concentrations of AG-NIL between VIDAS TB-IGRA and QFT Plus for the high-risk population. A *P*-value < 0.05 was considered statistically significant.

## RESULTS

### TB disease population

In this study, a total of 107 patient samples from three locations (Ouagadougou, Burkina Faso [101]; Newark, NJ, USA [4]; and Paris, France [2]) were included in the TB disease population. TB disease was confirmed by bacterial culture or PCR. These confirmed TB disease cases were used to compare the diagnostic performance of the VIDAS TB-IGRA and QFT-Plus tests. Three samples were excluded because no QFT-Plus results were available. Therefore, final data analysis was performed on 104 samples.

**Fig 3:**
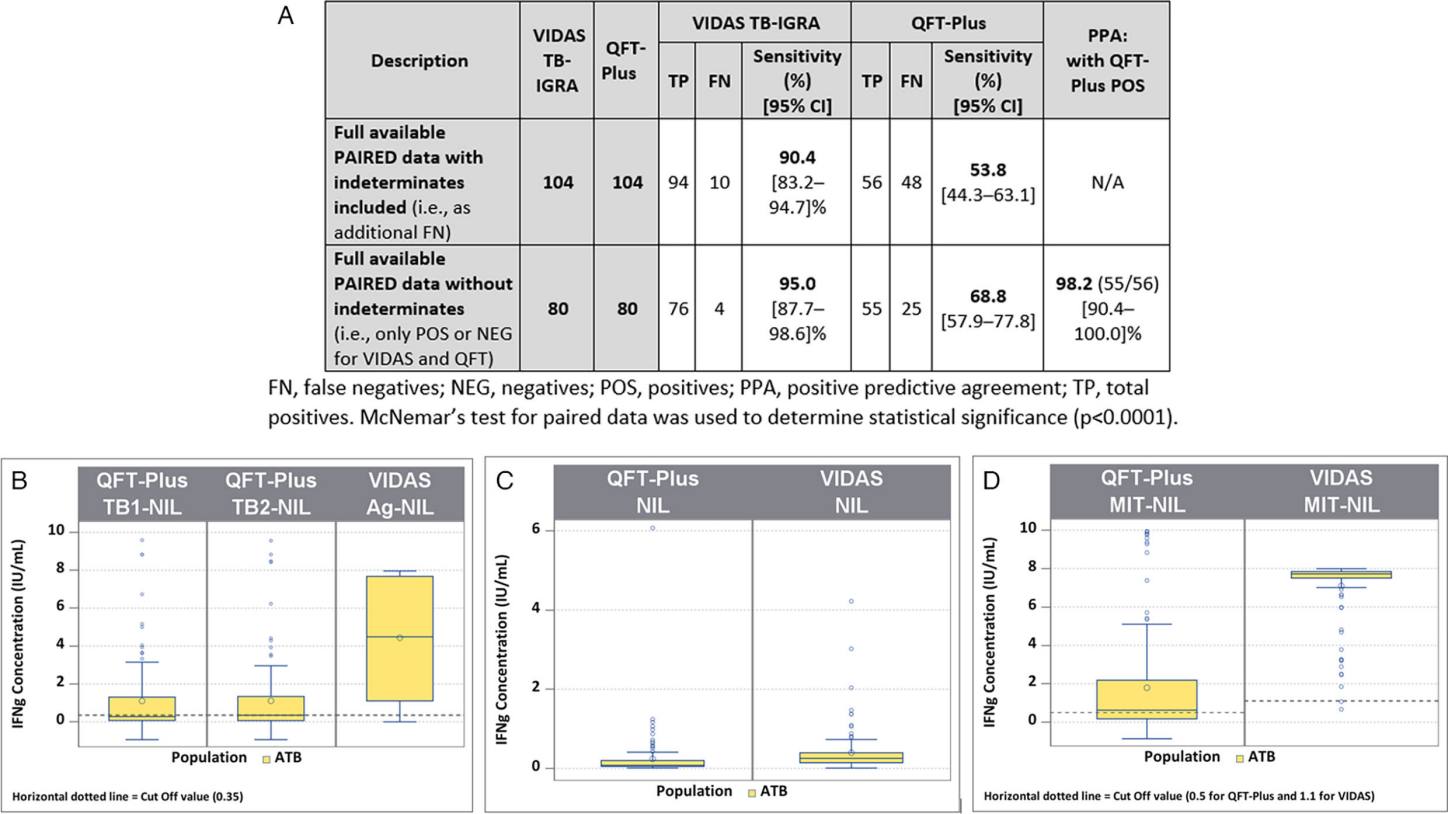
Comparison between the VIDAS TB-IGRA and QFT-Plus for the tuberculosis disease population. (A) Sample numbers, sensitivity, and positive predictive agreement. (B) Patient sample IFN-γ concentrations for the VIDAS TB-IGRA and QFT-Plus on AG-NIL intermediate result. (C) Patient sample IFN-γ concentrations for the VIDAS TB-IGRA and QFT-Plus on NIL intermediate result. (D) Patient sample IFN-γ concentrations for the VIDAS TB-IGRA and QFT-Plus on AG-NIL intermediate result. Nil, QFT-Plus negative control; NIL, VIDAS TB-IGRA negative control; Mitogen, QFT-Plus positive control; MIT-NIL, VIDAS TB-IGRA positive control; TB1/TB2, QFT-Plus mitogenic response samples; AG-NIL, VIDAS TB-IGRA mitogenic response sample. The dotted lines represent the sample threshold values for the respective assay. FN, false negatives; NEG, negatives; POS, positives; and TP, total positives. McNemar’s test for paired data was used to determine statistical significance (*P* < 0.0001).

In this TB disease population, only one indeterminate result was obtained with the VIDAS TB-IGRA, whereas 23 were obtained with the QFT-Plus. Two statistical analyses were performed to estimate sensitivity using equivalent data sets between the two tests (Fig. 3A).

The first analysis used results from 104 patients and included the indeterminate results as false negatives (94 true positives and 10 false negatives vs 56 true positives and 48 false negatives). In this analysis, the VIDAS TB-IGRA exhibited a sensitivity of 90.4%, whereas the QFT-Plus exhibited a sensitivity of 53.8% (*P* < 0.0001).

The second analysis excluded the indeterminate results (76 true positives and 4 false negatives vs 55 true positives and 25 false negatives) and used results from 80 patients. In this case, the VIDAS TB-IGRA exhibited a sensitivity of 95.0%, whereas the QFT-Plus exhibited a sensitivity of 68.8% (*P* < 0.0001). Furthermore, a 98.2% PPA was calculated between the two tests with this data set.

In the antigen response samples (AG-NIL vs TB1/2-NIL) and positive control samples (MIT-NIL vs Mitogen), the VIDAS TB-IGRA detected higher IFN-γ values than the QFT-Plus for this population (Fig. 3B through D). These higher values may have contributed to the improved detection capacity of the VIDAS TB-IGRA.

Additional information was collected from Burkina Faso patients who presented indeterminate results with low IFN-γ values for the positive control samples. The number of CD4^+^ T cells was analyzed in these patient samples to determine whether these low responses were due to a low number of CD4^+^ T cells responding to the mitogenic stimulation. The VIDAS TB-IGRA detected higher IFN-γ values in the AG-NIL and MIT-NIL samples compared with the QFT-Plus TB1/2 and Mitogen samples, regardless of the number of CD4^+^ T cells present (Fig. S1). Therefore, the differences observed between the two assays did not correlate with the number of CD4^+^ T cells. Many patients from the Burkina Faso location exhibited comorbidities and/or low body mass indexes, which may be an explanation for the low sensitivity of the QFT-Plus in these patients with TB disease (see supplemental Results).

### Low-risk population

A total of 117 healthy blood donor samples from the EFS in the Rhone-Alpes Auvergne region of France were included in the low-risk population for latent TB infection. In this population, the VIDAS TB-IGRA detected 6 positive and 111 negative samples. Likewise, the QFT-Plus detected 8 positive and 109 negative samples. No indeterminate results were obtained with either diagnostic test.

**Fig 4:**
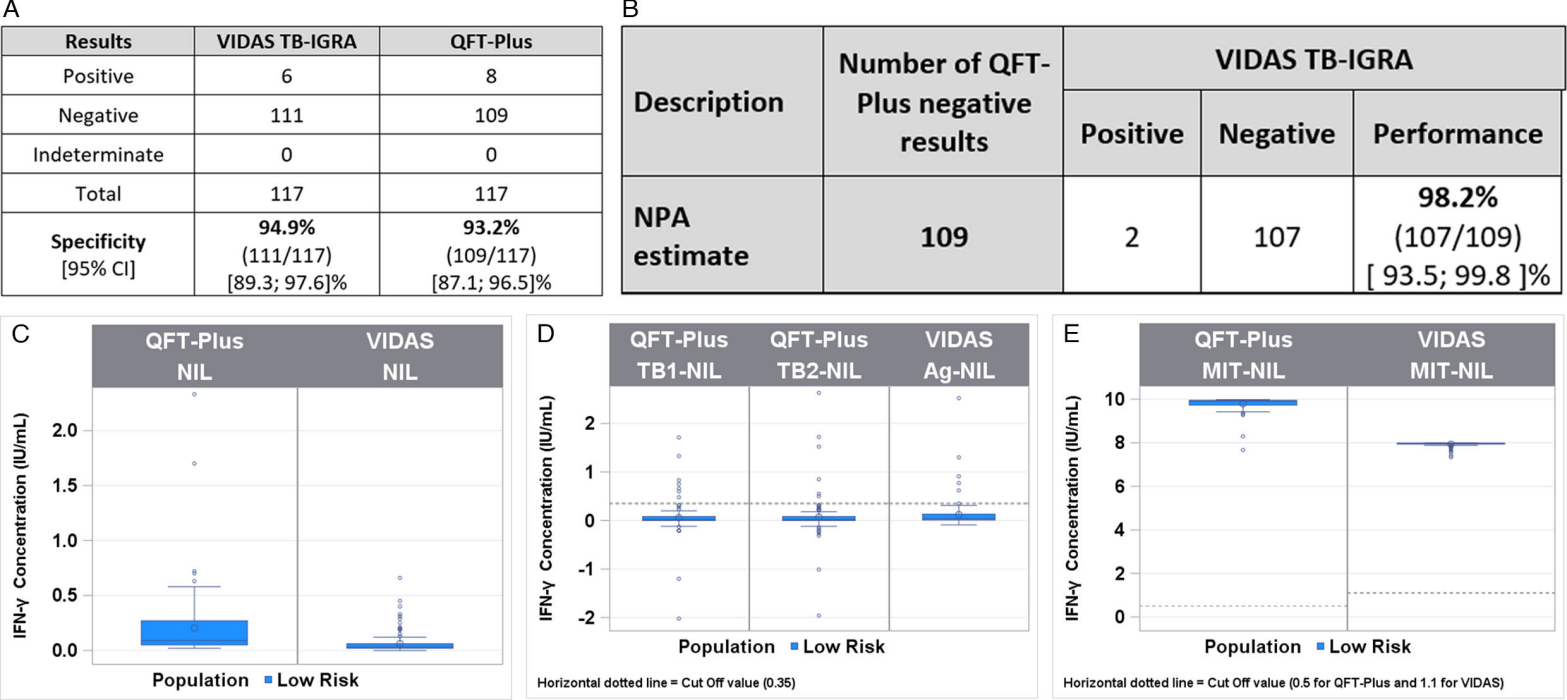
Comparison between the VIDAS TB-IGRA and QFT-Plus for the low-risk population. (A) Specificity estimates for the two assays. (B) Sample numbers and NPA. (C) Patient sample interferon-γ concentrations for the VIDAS TB-IGRA and QFT-Plus on AG-NIL intermediate result. (D) Patient sample interferon-γ concentrations for the VIDAS TB-IGRA and QFT-Plus on NIL intermediate result. (E) Patient sample interferon-γ concentrations for the VIDAS TB-IGRA and QFT-Plus on MIT-NIL intermediate result. Nil, QFT-Plus negative control; NIL, VIDAS TB-IGRA negative control; Mitogen, QFT-Plus positive control; MIT-NIL, VIDAS TB-IGRA positive control; TB1/TB2, QFT-Plus mitogenic response samples; and AG-NIL, VIDAS TB-IGRA mitogenic response sample. The dotted lines represent the sample threshold values for the respective assay.

These healthy blood donors were from a country with a very low prevalence of TB, and participants with any TB history were excluded from sample recruitment. Therefore, this population presented an extremely low risk of infection and was used to evaluate the specificity of the two tests. The VIDAS TB-IGRA exhibited a specificity of 94.9%, and the QFT-Plus exhibited a similar specificity of 93.2% (Fig. 4A).

We also observed a strong NPA of 98.2% between the VIDAS TB-IGRA and QFT-Plus, further indicating that the two tests exhibited similar diagnostic performance in this low-risk population (Fig. 4B). Furthermore, the VIDAS TB-IGRA detected low basal IFN-γ values in the AG-NIL samples, suggesting that the test maintained its specificity despite an improved detection capacity for latent TB infection (Fig. 4C through E).

### High-risk population

**Fig 5:**
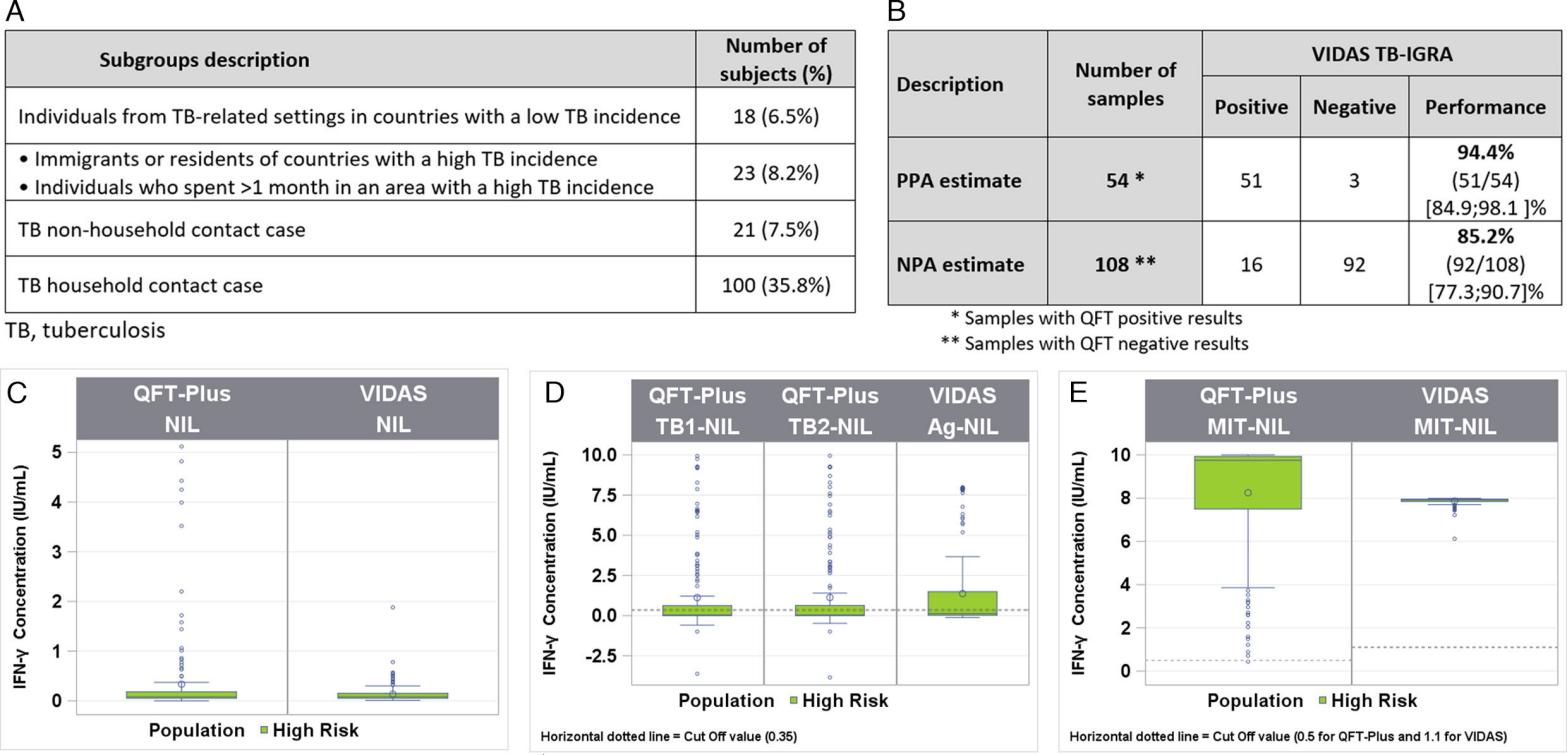
Comparison between the VIDAS TB-IGRA and QFT-Plus for the high-risk population. (A) Subgroups’ description. (B) Sample numbers, PPA, and NPA. (C) Patient sample interferon-γ concentrations for the VIDAS TB-IGRA and QFT-Plus for AG-NIL intermediate result. (D) Patient sample interferon-γ concentrations for the VIDAS TB-IGRA and QFT-Plus for NIL intermediate result. (E) Patient sample interferon-γ concentrations for the VIDAS TB-IGRA and QFT-Plus for MIT-NIL intermediate result. Nil, QFT-Plus negative control; NIL, VIDAS TB-IGRA negative control; Mitogen, QFT-Plus positive control; MIT-NIL, VIDAS TB-IGRA positive control; TB1/TB2, QFT-Plus mitogenic response samples; and AG-NIL, VIDAS TB-IGRA mitogenic response sample. The dotted lines represent the sample threshold values for the respective assay. A Wilcoxon’s test for paired data was used to determine statistical significance between VIDAS TB-IGRA and QFT Plus for the response AG-NIL (*P* < 0.0001 for both TB1 and TB2).

A total of 162 patient samples from two locations (Ouagadougou, Burkina Faso [90]; Newark, NJ, USA [72]) were included in the high-risk population for latent TB infection. The Burkina Faso samples were all contact cases. In contrast, the Newark, NJ samples consisted of contact cases, immigrants from countries with a high prevalence of TB disease, and healthcare workers with an increased risk of exposure (Fig. 5A). Of these samples, 67 were determined as positive and 95 were determined as negative by the VIDAS TB-IGRA, whereas 54 were determined as positive and 108 were determined as negative by the QFT-Plus. No indeterminate results were obtained with either diagnostic test.

On qualitative interpretation, we observed a strong PPA of 94.4% but a lower NPA of 85.2% (Fig. 5B) between both tests. The quantitative results showed that the VIDAS TB-IGRA detected higher IFN-γ values (*P* < 0.0001), resulting in the identification of more positive samples than the QFT-Plus (Fig. 5C through E). This discrepancy was independently observed at the two locations that recruited the majority of the high-risk patients at a similar level (13.3% and 9.7% of the total results were discrepant in Burkina Faso and Newark, NJ, respectively). The discrepant results for this high-risk population are provided in Table 2.

**TABLE 2:** Discrepant results on high-risk population

Sample ID	Clinical context	VIDAS TB-IGRA				QFT-Plus				
		NIL	Ag-NIL	MIT-NIL	Interp.	NIL	TB1-NIL	TB2-NIL	MIT-NIL	Interp.
12	Healthcare worker	0.14	0.40	7.86	P	0.25	0.32	0.33	9.75	N
7	Immigrant from a country with a high incidence of TB	0.21	0.75	7.79	P	0.5	−0.11	−0.03	9.5	N
3	Immigrant from a country with a high incidence of TB	0.02	1.33	7.98	P	0.25	0.03	0.06	9.75	N
18	Immigrant from a country with a high incidence of TB	0.19	3.55	7.81	P	0.21	0.22	0.25	9.79	N
9	Immigrant from a country with a high incidence of TB	0.02	0.57	7.98	P	0.12	0.18	0.21	9.88	N
19	Contact with a sick person	0.11	0.59	7.89	P	0.22	−0.07	−0.08	9.78	N
2	Contact with a sick person	0.55	3.02	7.45	P	0.03	0.08	0.15	9.97	N
16	Contact with a sick person within household	0.16	1.18	7.84	P	0.07	0.15	0.34	9.93	N
6	Contact with a sick person within household	0.15	1.43	7.85	P	0.06	−0.02	0.04	9.94	N
10	Contact with a sick person within household	0.21	0.41	7.79	P	0.06	0.10	0.04	9.94	N
13	Contact with a sick person within household	0.22	0.45	7.78	P	0.03	0.02	0.00	9.97	N
4	Contact with a sick person within household	0.30	0.58	7.70	P	0.07	0.12	0.06	6.9	N
14	Contact with a sick person within household	0.53	0.43	7.47	P	0.12	0.04	0.01	3.16	N
15	Contact with a sick person within household	0.06	1.20	7.94	P	0.02	0.22	0.09	3.52	N
5	Contact with a sick person within household	0.07	0.74	7.93	P	0.03	0.07	0.08	7.64	N
17	Contact with a sick person within household	0.08	1.59	7.92	P	0.14	0.23	0.26	9.86	N
8	Contact with a sick person within household	0.29	0.26	7.71	N	0.06	1.09	0.97	9.5	P
11	Contact with a sick person within household	0.05	0.04	7.95	N	0.02	0.88	1.07	2.6	P
1	Contact with a sick person within household	0.08	0.32	7.92	N	0.05	0.43	0.37	8.86	P

^*a*^ P, positive; N, negative.

## DISCUSSION

Here, we provide the first evaluation of the diagnostic performance of the VIDAS TB-IGRA in detecting both latent TB infection and disease in three patient populations compared with the widely used QFT-Plus.

In the TB disease population, the VIDAS TB-IGRA exhibited high sensitivity compared with international gold standard methods. Furthermore, VIDAS TB-IGRA outperformed QFT-Plus by exhibiting higher sensitivity. The enhanced sensitivity of this test was most likely due to its capacity to provide a positive interpretation in samples that had an indeterminate or false-negative result with the QFT-Plus. The better performance of VIDAS TB-IGRA may be multifactorial, encompassing optimized reagent formulations, tailored stimulation conditions, and optimized testing protocols. The stimulation conditions defined for the product may contribute to favorable conditions for cellular secretion of IFN-γ by T cells, thus allowing a better detection of both specific and non-specific responses.

The QFT-Plus exhibited a lower sensitivity than previously indicated by the manufacturer and also yielded a large number of indeterminate results (9). These indeterminate results were most likely not due to technical issues, as all hospital personnel were thoroughly trained to ensure that all procedures were performed according to the manufacturer’s recommended guidelines and instructions. Furthermore, correct sample processing was validated as samples from both TB disease and high-risk populations were simultaneously assayed in the same microplates. No indeterminate results were obtained for the high-risk population with the QFT-Plus.

As the number of indeterminate results with QFT-Plus was significantly higher than in most other studies published, we investigated further. Considering the period of recruitment for this study, which was between October 2019 and February 2020, it may be possible that there may have been concomitant SARS-CoV infections that were not yet suspected. Studies have shown that patients hospitalized due to SARS-CoV-2 infection demonstrated lower IFN-γ production in the Mitogen-Nil controls associated with a lower number of peripheral blood T-lymphocytes (10). The number of indeterminates in the case of a SARS-CoV infection can therefore increase significantly (11, 12).

In addition, the relatively high frequency of indeterminate results is likely to be associated with patients (immunocompromised groups) involved in the study. Indeed, a recent study done in Burkina Faso reported that TB patients are characterized by remarkable lymphopenia, monocytosis, thrombocytosis, and hypochromic microcytic anemia. These hematological defects could increase indeterminate results of IGRAs (13). A regression predictive model for QuantiFERON-TB Gold Plus indeterminate results in immunosuppressed patients found that lymphopenia, hypoalbuminemia, and decreased estimated glomerular filtration rate were significant risk factors for indeterminate results (14).

The low sensitivity of the QFT-Plus in detecting TB disease cases is documented (15–17), particularly in countries with a high prevalence of *M. tuberculosis* infection and other comorbidities (18), such as Burkina Faso, which was one of our study sites. Additionally, there may be a potential correlation between the low sensitivity of the QFT-Plus and a high frequency of comorbidities, particularly parasitic infections and fecal yeast (19–21(21) in these patients. Because the sensitivity of the QFT-Plus has been previously determined in countries with a low prevalence of *M. tuberculosis* infection and other comorbidities, including the US, Japan, and Australia (9), the low sensitivity and high number of indeterminate results obtained with the QFT-Plus in this study may be due to the particularly poor health status of the Burkina Faso study participants. However, this does not seem to influence the VIDAS TB-IGRA results. Further investigations are needed to compare the performance of the two tests in various geographical locations and under different contexts.

In the low-risk population, the VIDAS TB-IGRA exhibited a high NPA with the QFT-Plus results, thus demonstrating that the two tests had a similar performance in correctly identifying negative samples. Additionally, the VIDAS TB-IGRA detected low basal IFN-γ values in the AG-NIL samples, further indicating that the specificity of the test was maintained despite its improved detection capacity.

It is difficult to evaluate the capacities of the IGRAs within a high-risk population without a gold standard method of reference. The VIDAS TB-IGRA demonstrated a high PPA with the QFT-Plus results, indicating its comparable performance in detecting latent TB infection in this patient group. The lower NPA result comes as no surprise, as we have demonstrated a higher sensitivity on the culture-confirmed TB disease population. We have also shown previously that the specificity of the test is adequate on a low-risk population in a low-prevalence country, France. Our hypothesis, therefore, is that the higher sensitivity on a TB disease population may also extend to the high-risk population.

Our study had some limitations. The number of participants was limited, and all participants in the low-risk population were derived from a single location. Additional multi-center, prospective studies are warranted to support our results. Furthermore, the system’s alarm thresholds were not properly adjusted for the new assay, which led to unnecessary alarms being triggered (see system errors in Fig. 1A through C). After the study was completed, the alarm thresholds were optimized to better suit the assay, and this resolved the problem. New data should be generated to prove the efficiency of the corrective solution.

In conclusion, the VIDAS TB-IGRA exhibits higher sensitivity than a currently used IGRA while maintaining comparable specificity. In addition, its fully automated system allows for quick and accurate delivery of single-patient results. These characteristics point to the VIDAS TB-IGRA as an improved aid in the diagnosis of individuals infected with *Mycobacterium tuberculosis* (latent infection or disease).

## SUPPLEMENTAL MATERIAL

Supplemental material jcm.01641-24-s0001.docx Additional information associated to CD4 cell count and patients comorbidities.

ASM does not own the copyrights to Supplemental Material that may be linked to, or accessed through, an article. The authors have granted ASM a non-exclusive, world-wide license to publish the Supplemental Material files. Please contact the corresponding author directly for reuse.
