# Supplementary material for: Preliminary performance of the VIDAS TB-IGRA as an aid in the diagnosis of individuals infected with Mycobacterium tuberculosis
Source: J Clin Microbiol. 2025 May 14;63(6):e01641-24. doi: 10.1128/jcm.01641-24 (PMC12153259; doi:10.1128/jcm.01641-24)
Supplement: Supplemental material — Additional information associated to CD4 cell count and patients comorbidities. [file jcm.01641-24-s0001.docx]

**SUPPLEMENTAL MATERIAL**

**Analysis of CD4^+^ T-cell counts in patient samples from the TB disease population in Burkina Faso**

Low TB1, TB2 antigen and MIT-NIL IFN-γ responses resulted in indeterminate QFT-Plus results in the TB disease population. Therefore, the number of CD4^+^ T cells was analyzed in the TB disease patient samples from the Burkina Faso location. Higher IFN-γ values in the AG-NIL and MIT-NIL samples were detected with the VIDAS**®** TB-IGRA compared with the QFT-Plus TB1, TB2 and MIT-NIL samples, respectively, regardless of CD4^+^ T-cell counts (Supplemental Figure 1). Therefore, no correlation between the number of CD4^+^ T cells present in the patient samples and the extent of the IFN-γ responses was observed, suggesting that the higher sensitivity of the VIDAS**®** TB-IGRA was most likely due to design optimization rather than the number of CD4^+^ T cells in the patient samples.

**Characteristics of Burkina Faso patients with TB disease**

Various characteristics were observed in the patients from the Burkina Faso location (Supplemental Table 1). Specifically, there was a high presence of parasitic infections and fecal yeast in these patients. Of the patients with TB disease, approximately 25% had parasitic infections and approximately 52% had yeast in their fecal samples. Likewise, approximately 21% of the high-risk patients had parasitic infections and approximately 59% exhibited yeast in their fecal samples. Additionally, nearly half (49%) of the Burkina Faso patients with TB disease had low BMIs (<18.5 kg/m^2^), possibly indicating malnutrition. Thus, the low sensitivity of the QFT-Plus in detecting TB infection and disease in these particular patient demographics may be at least partially attributed to the poor health status of these patients.

**Supplemental Figure 1.** Analysis of CD4^+^ T-cell densities in patient samples from the tuberculosis disease population in Burkina Faso. **A.** Interferon-γ (IFN-γ) concentrations in patient samples stratified by CD4^+^ T-cell numbers for AG-NIL and MIT-NIL results. **B.** Percentage of patients with tuberculosis disease according to CD4^+^ T-cell numbers.


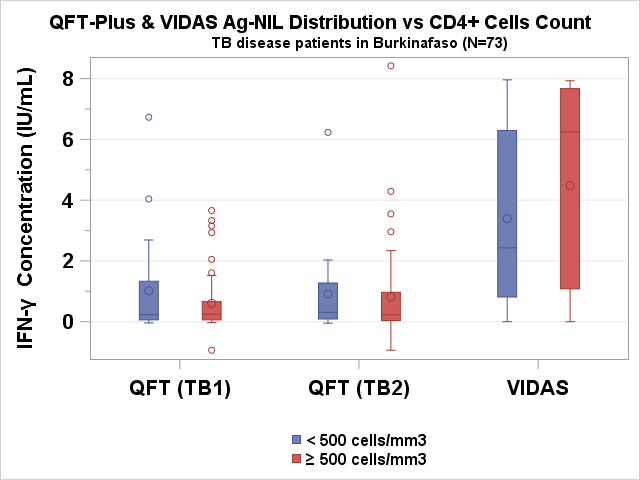


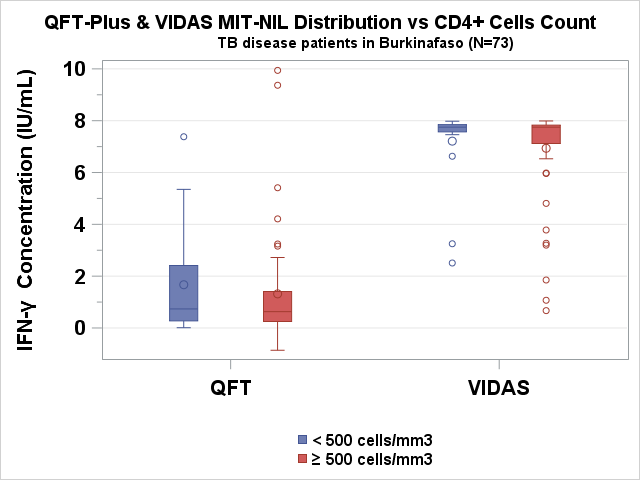


| Population recruited | Tuberculosis disease (Burkina Faso) |
| --- | --- |
| Study population, N (%) | 101 (100%) |
| Level of CD4^+^ T cells, N (%) |  |
| <500 cells/mm^3^ | 20 (19.8%) |
| >500 cells/mm^3^ | 53 (52.5%) |
| Unknown | 28 (27.7%) |

**SUPPLEMENTAL TABLES**

**Supplemental Table 1.** Demographics and additional information collected from patients included in the study.

| **Populations recruited** | **Low-risk** | **High-risk** | **Tuberculosis disease** |
| --- | --- | --- | --- |
| Study population, N (%) | 117 (100%) | 162 (100%) | 107 (100%) |
| Age in years, median (range) | 36 (18–67) | 37 (6–73) | 37 (13–82) |
| Sex, N (%) |  |  |  |
| Male | 70 (59.8%) | 73 (45.1%) | 86 (80.4%) |
| Female | 47 (40.2%) | 89 (54.9%) | 21 (19.6%) |
| Number provided by location, N (%) |  |  |  |
| Ouagadougou, Burkina Faso | - | 90 (55.5%) | 101 (94.4%) |
| Newark, NJ, USA | - | 72 (44.5%) | 4 (3.7%) |
| Paris, France | - | - | 2 (1.9%) |
| Lyon, France | 117 (100%) | - | - |
| Race, N (%) |  |  |  |
| Ouagadougou, Burkina Faso | - | 90 (56%) Africans and | 101 (94.4%) Africans |
| Newark, NJ, USA | - | 72 (44%) Americans, including 9 Asians, 21 Blacks/African Americans, and 42 Whites | 4 (3.7 %) Americans, including 3 Whites and 1 Black/African American |
| Paris, France | - | - | 2 (1.9%) Africans |
| Lyon, France | - | - | - |
| Ethnicity (US ONLY) | - | 72 (44%) Americans, including 42 Hispanics/Latinos and 30 Non-Hispanics/Latinos | 4 (4%) Americans, including 3 Hispanics/Latinos and 1 Non-Hispanic/Latino |
| Location of tuberculosis disease, N (%) |  |  |  |
| Pulmonary | - | - | 104 (97.2%) |
| Extra-pulmonary | - | - | 3* (2.8%) |
| ** 1 Bone, 1 cervical, 1 nodular* |  |  |  |
| HIV status, N (%) |  |  |  |
| Positive | - | 2 (1%) | - |
| Negative | 117 (100%) | 100 (62%) | 107 (100%) |
| Unknown | - | 60 (37%) | - |
| BCG vaccination, N (%) |  |  |  |
| Yes | - | 114 (70%) | 61 (57%) |
| No | - | 35 (22%) | 8 (7.5%) |
| Unknown | 117 (100%) | 13 (8%) | 38 (35.5%) |
| Diabetes, N (%) |  |  |  |
| Yes | - | 8 (5%) | 1 (1%) |
| No | 117 (100%) | 154 (95%) | 106 (99%) |
| Autoimmune diseases, N (%) |  |  |  |
| Yes | - | 3 (2%) | 1 (1%) |
| No | 117 (100%) | 159 (98%) | 106 (99%) |
| Infectious diseases, N (%) |  |  |  |
| Yes | - | 2 ( 1%) | 3 (3%) |
| No | 117 (100%) | 160 (99%) | 104 (97%) |
| Other pathologies, N (%) |  |  |  |
| Yes | - | 24 (15%) | 13 (12%) |
| No | 117 (100%) | 138 (85%) | 94 (88%) |
| Antibiotic treatments, N (%) |  |  |  |
| Yes | - | 3 (2%) | 106 (99%) |
| No | 117 (100%) | 159 (98%) | 1 (1%) |
| Immunosuppressive treatments, N (%) |  |  |  |
| Yes | - | 1 (1%) | - |
| No | 117 (100%) | 161 (99%) | 107 (100%) |
| Pain treatment (analgesics), N (%) |  |  |  |
| Yes |  | 5 (3%) | 11 (10.3%) |
| No | 117 (100%) | 157 (97%) | 96 (89.7%) |
| Other treatments, N (%) |  |  |  |
| Yes | - | 21 (13%) | 14 (13%) |
| No | 117 (100%) | 141 (87%) | 93 (87%) |
| TST, N (%) |  |  |  |
| Yes | - | 35 (22%) | 1 (1%) |
| No | 18 (15%) | 123 (76%) | 103 (96%) |
| Unknown | 99 (85%) | 4 (2%) | 3 (3%) |
